# Realising Meaningful Human Control Over Automated Driving Systems: A Multidisciplinary Approach

**DOI:** 10.1007/s11023-022-09608-8

**Published:** 2022-07-28

**Authors:** Filippo Santoni de Sio, Giulio Mecacci, Simeon Calvert, Daniel Heikoop, Marjan Hagenzieker, Bart van Arem

**Affiliations:** 1 Delft University of Technology, Delft, The Netherlands; 2 Donders Institute, Radboud University, Nijmegen, The Netherlands

**Keywords:** Self-driving cars, Responsibility gap, Meaningful human control, Driver's psychology, Core components of automated driving systems

## Abstract

The paper presents a framework to realise “meaningful human control” over Automated Driving Systems. The framework is based on an original synthesis of the results of the multidisciplinary research project “Meaningful Human Control over Automated Driving Systems” lead by a team of engineers, philosophers, and psychologists at Delft University of the Technology from 2017 to 2021. Meaningful human control aims at protecting safety and reducing responsibility gaps. The framework is based on the core assumption that human persons and institutions, not hardware and software and their algorithms, should remain ultimately—though not necessarily directly—in control of, and thus morally responsible for, the potentially dangerous operation of driving in mixed traffic. We propose an Automated Driving System to be under meaningful human control if it behaves according to the relevant reasons of the relevant human actors (tracking), and that any potentially dangerous event can be related to a human actor (tracing). We operationalise the requirements for meaningful human control through multidisciplinary work in philosophy, behavioural psychology and traffic engineering. The tracking condition is operationalised via a proximal scale of reasons and the tracing condition via an evaluation cascade table. We review the implications and requirements for the behaviour and skills of human actors, in particular related to supervisory control and driver education. We show how the evaluation cascade table can be applied in concrete engineering use cases in combination with the definition of core components to expose deficiencies in traceability, thereby avoiding so-called responsibility gaps. Future research directions are proposed to expand the philosophical framework and use cases, supervisory control and driver education, real-world pilots and institutional embedding

## Automated Driving Systems and Responsibility

Automated driving systems (henceforth ADS) are widely expected to yield strong societal and economic benefits, by increasing road capacity, reducing congestion, reducing crashes and travel time, improving fuel efficiency, productivity and parking (Fagnant & Kockelman, 2015; Milakis et al, 2017). According to the widely used SAE J3016 taxonomy (SAE, 2018), Driving Automation Systems are defined as any hardware and software system or feature that are collectively capable of performing the entire (Automated Driving Systems) or parts (Driver Support Features) of the dynamic driving task of a road vehicle on a sustained basis. The operation of Driving Automation Systems may be restricted to a specific Operational Design Domain. For an excellent overview on Driving Automation developments, examples, issues and directions, we refer to Shladover (2018).

**Fig. 1:**
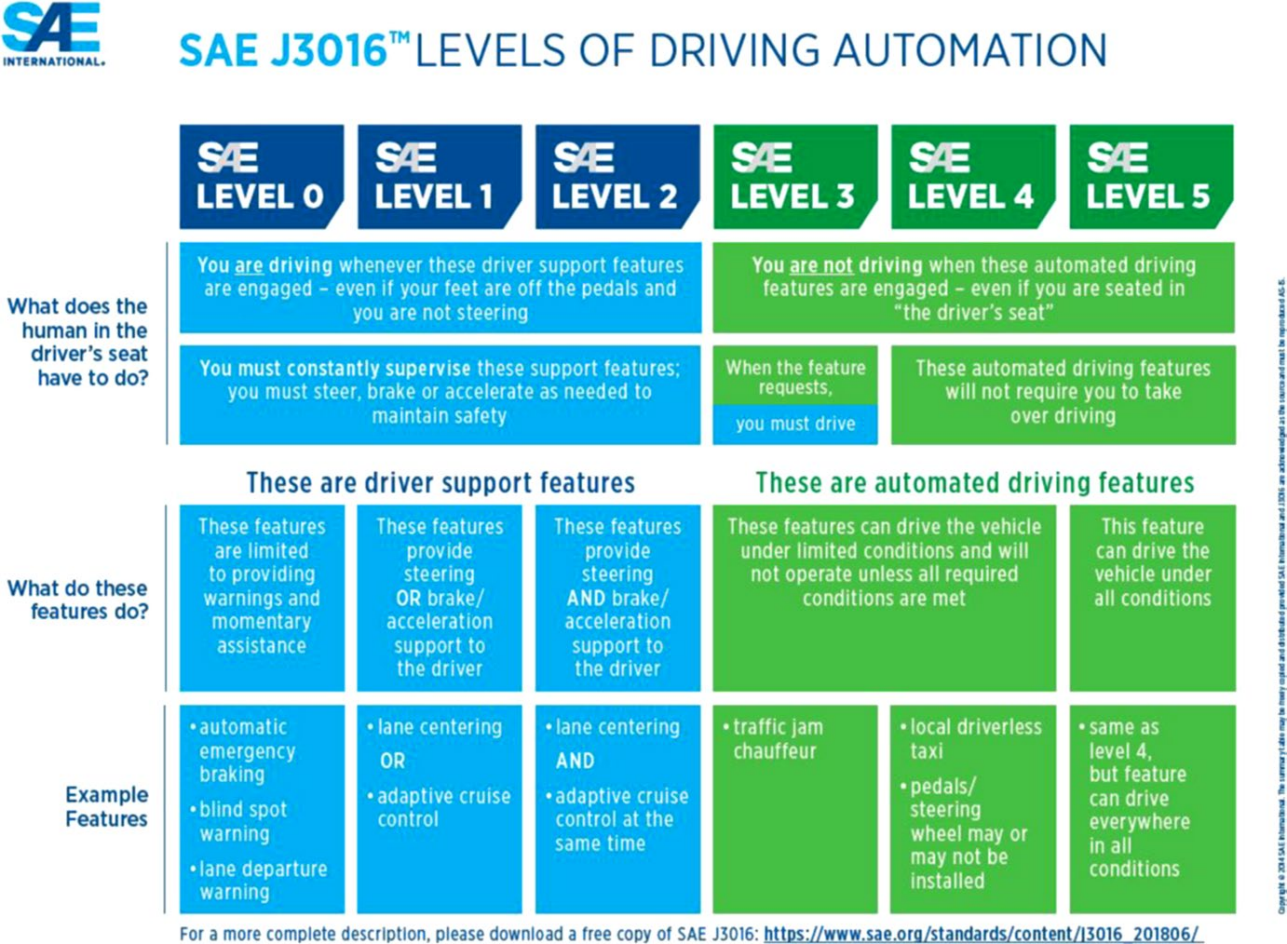
SAE J3016 levels of driving automation

To provide guidance and common terminology of Driving Automation Systems, Fig. 1 summarises 5 levels of driving automation and their interaction with the human in the driver’s seat. At level 0–2, the human is considered to still drive the vehicle and to constantly supervise the support features and intervene as needed. At levels 3–5 the human is not considered to be driving. At level 3, the driver must resume operation of the road vehicle when the automated driving requests. Therefore, up to level 3 the human in the driver’s seat is required to be skilled in interacting with the automated vehicle. This leads to high requirements on the capability of the automated driving features to correctly function and communicate with the human. It also raises concerns about the ability of humans to successfully acquire skills to interact with automation features beyond those acquired in current driving education programs and driving experience (Kyriakidis et al, 2019).

Early ethical and social science reflections on the introduction of ADS have focused on a series of fictional scenarios in which an ADS faces an emergency situation in which a crash is unavoidable and the only choice open is one between hitting two or more different “targets” (Nyholm, 2018 for a summary). The ethical question is posed, as to how the ADS should be programmed to behave in these and similar circumstances. While these fictional scenarios have captured much of the public attention, a wider set of ethical issues has been discussed in the literature, ranging from issues of risk and safety (Goodall, 2016), distributive justice issues (Mladenovic & McPherson, 2016) rights and inequalities (Liu, 2017), human control (Mecacci & Santoni de Sio, 2020), responsibility (Hevelke & Nida-Rümelin, 2014) the political dimension of vehicle automation (Himmelreich, 2019; JafariNaimi, 2018; Stilgoe, 2017).

This paper presents a comprehensive, original synthesis of the results of the multidisciplinary research project “Meaningful Human Control over Automated Driving Systems” lead by a team of engineers, philosophers, and psychologists—the authors of this paper—at Delft University of the Technology from 2017 to 2021. The project addresses one specific ethical and philosophical issue: what kind of control and responsibility can and should different human actors in the network of ADS maintain over ADS. The relevance of the above question is grounded in two considerations. First, no matter how many tasks will be shifted from the driver to the ADS, the human element will never be eliminated from the equation, be it in the form of persons controlling the driving systems or just designing, regulating, or interacting as road users with them. So, unlike sometimes assumed, ADS will not necessarily reduce the demands on human actors, but they will rather change and redistribute them, or even create new ones. If this transition is not properly managed, so-called “responsibility gaps” may emerge. Responsibility gaps are situations in which some (undesirable) outcome occurs but in which it is not clear who is/was supposed to prevent it from happening, and who is morally and legally responsible (Matthias, 2004; Santoni de Sio & Mecacci, 2021). Therefore, new forms of control and responsibility will have to be designed for drivers and others stakeholders. Second, as a matter of fact, rather than a quick migration to fully automated vehicles (level 5), two main emerging intermediate steps towards driving automation seem realistic in the coming 10–15 years, namely (1) partial autonomy, such as driving automation features, potentially leading to dual mode vehicles that can be driven either manually or in automated mode and (2) supervised automated such as computer controlled ‘pods’ continuously monitored by a (possibly remote) human supervisor, while other combinations of mode may also exist.

The approach of that project and this paper is rooted in the generic ideal of “meaningful human control (MHC)” over autonomous systems as originally presented by (Santoni de Sio and van den Hoven 2018). Applied to ADS, our core fundamental assumption is that human persons and institutions, not hardware and software and their algorithms should remain ultimately, once all control chains are considered—and though not necessarily directly—in control of, and thus morally responsible for, the potentially dangerous operation of driving in mixed traffic. We consider meaningful human control to be crucial to protect the right to safety and the principle of human accountability as fundamental for Responsible Innovation in ADS. Given the moral responsibility of designers to protect and promote human safety (Nihlén Fahlquist, 2009), we claim that the introduction of ADS should aim to both reduce the number of (fatal) accidents currently happening with manual driving systems and to avoid the creation of new serious threats to human safety (Bonnefon et al., 2020). But this, as we argue, cannot be granted if ADS are not under some relevant form of human control and there are gaps in responsibility. When this happens, the relevant human actors may not be sufficiently able, motivated and willing to prevent undesired outcomes (Elish, 2019; Flemisch et al., 2017). In addition, we consider human responsibility also to be important in order to prevent legitimate discontent among victims of accidents and distrust towards technology more generally (Danaher, 2016).

In order to define and operationalise the principle of MHC in relation to ADS, in line with the principles of Responsible Innovation and Value-sensitive Design (van den Hoven, 2007) we take a multidisciplinary and design-oriented approach along three interrelated disciplinary research directions. From a philosophical perspective, we develop a definition of MHC over ADS and explore the relationship between MHC and moral and legal responsibility for the behaviour of ADS. From a behavioural science perspective, we analyse the impact of the introduction of ADS on human abilities and motivation, and the abilities and preferences that human agents should possess in order to maintain an automated driving system under meaningful human control. From a traffic engineering perspective, we study how MHC over ADS can be achieved in dynamic traffic environments involving interactions between human and artificial agents and improve the efficiency of a road system while at the same time maintaining the system under MHC.

This paper proposes an original synthesis of the advances that were made in each of these research directions in the articles previously published by the authors. The novelty of this contribution lies in the combination of these advances rather than the contributions in each of the directions. For the latter the reader is referred to additional publications.

## The Philosophical Background: Responsibility Gaps and Meaningful Human Control

### Responsibility Gaps

Responsibility gaps are situations where responsibility is difficult or impossible to attribute to one or more human agents due to the presence of automated agency in a sociotechnical system. Despite the notion of responsibility gap has been widely used in different contexts, its nature and scope has remained insufficiently analysed. In particular, this notion is often used without accounting for the complexity of the concept of responsibility. It was Andreas Matthias (2004) who originally discussed the potential impact of artificial intelligence (AI) and more specifically machine learning (in his words: learning automata) on attribution of moral culpability. In a nutshell: legitimate attribution of moral culpability for untoward event requires some form of prediction and control by human actors; but the interaction with machine learning systems may make this prediction and control very difficult. Therefore, machine learning may increase complexity to legitimately attribute moral culpability to human actors for their actions (whenever these are mediated by machine learning systems).

What Matthias described is thus the risk of gaps in *moral culpability* caused by (the unpredictability of) machine learning. (Sparrow, 2007) and other have shared Matthias’ concern about a possible culpability gap in relation to learning autonomous weapon systems. The risks of culpability gaps in relation to autonomous technologies more generally have also been discussed from a legal perspective (Calo, 2015; Pagallo, 2013). The discussion on responsibility gap has now gone far beyond the original formulation by Matthias. (Mittelstadt et al., 2016) have argued that gaps may emerge not only due to the learning capacities of AI but mainly due to the opacity, complexity and unpredictability of present-day AI systems. Similar considerations are also behind the literature on so-called gaps in “transparency” and “explainability” of AI systems (Doran et al., 2017) and their moral (Coeckelbergh, 2020) and legal implications (Edwards & Veale, 2017; Noto La Diega, 2018; Wachter et al., 2017). Some authors have argued against the existence, relevance, or novelty of AI-induced responsibility gaps (Simpson & Müller, 2016; Tigard, 2020) while others have proposed general principles to address (some aspects of) the responsibility gaps, by focusing on the (new) roles of human agents in the systems of which AI is a part (Nyholm, 2018; Santoro et al., 2008).

In contrast with deflationist approaches denying the relevance of responsibility gaps with AI, and by taking stock of recent literature on the responsibility gap in philosophy, law, and ethics of technology, Santoni de Sio & Mecacci (2021), have recently proposed a classification of responsibility gaps. They identify four kinds of gaps: in culpability (blameworthiness), gaps in moral accountability (capacity to understand and explain to others the behaviour of a system of which one is part), gaps in public accountability (capacity of public officials to understand and explain to some relevant forum the behaviour of a system they are responsible for); and gaps in active responsibility (capacity to comply with one’s obligations in relation to the behaviour of technological systems). They argue that all of these gaps must be avoided as they affect the realisation of the (moral) value of the four types of responsibility. With the possible exception of moral accountability, these gaps have both a moral and a legal dimension, which often overlap but never fully coincide (moral and legal culpability; public accountability as a moral or a legal duty; moral and legal obligation to ensure that a product does not cause harm/produce benefits).

Santoni de Sio & Mecacci (2021) also clarify that responsibility gaps may be caused by different sources, some of which are old, i.e. the complexity and multi-agential nature of social and technical systems, some new, i.e. the data-driven learning features of present-day AI; some more technical, i.e. the intrinsic opacity of algorithmic decision-making, some more political and economical, i.e. the implicit privatisation of public agencies and spaces; some more moral and societal, i.e. the engineers’ and other actors’ lack of awareness and/or capacity to comply with their (new) moral, legal, societal obligations. Correspondingly, they criticise attempts to address responsibility gaps by only looking at one of their dimensions, for instance reducing opacity via more “explainable AI” (Doran et al., 2017) or filling liability gaps via new legal arrangements, such as legal personhood for AI agents (Delvaux, 2017). They advocate for a more comprehensive approach, one that may allow to address the responsibility gap in its different dimensions. We endorse both their proposed general project as well as their specific suggestion that such a project may be realised by developing the approach to “meaningful human control” developed by Santoni de Sio and Van den Hoven (2018).

### Meaningful Human Control

The transition to networked and AI-based systems may create control problems, that are part of a general problem with the interactions of human controllers with AI and intelligent systems. Human controllers of intelligent systems can lose track of their role in the control chain, ending up not being able to effectively steer the system in the desired direction though remaining, technically speaking, “in-the-loop”, or legally liable for it. This is due to several factors, from the systems’ fast and resolute decision-making capacity to the huge amount of information at their disposal. The ethical and political concern of human persons and institutions losing control on the behaviour of AI-based systems has been particularly strong in relation to so-called lethal autonomous weapon systems (Human Right Watch, 2015). To address these concerns, different stakeholders converged towards the idea that a more meaningful form of control should be granted over AI and intelligent technologies. Multiple accounts of meaningful human control (MHC henceforth) have been recently produced in relation to autonomous weapon systems (see (Ekelhof, 2019)). These mostly consist of sets of standards to promote a legally, ethically and societally acceptable form of human control, typically by a designated operator of an AI-based weapon system, like a military commander. This conception of control is similar to the one present in the Geneva Convention on road traffic of 1949 and the Vienna Convention on road traffic 1968, in that it defines control in terms of the possibility of one operator to directly steer the behaviour of a technical device or system (Vellinga, 2019). It is also close to the idea of “controllability” as presented in safety standards for the automotive industry such as the ISO 26262.

However, the philosophical debate over control of complex socio-technological (AI) systems is certainly broader than that. On the one hand, Bostrom and others have famously addressed the question, to what extent and under which conditions we as a society can control the future development of AI in such a way that this remains aligned with some relevant human goals, or remains “human-compatible” (Bostrom, 2014; Flemisch, 2008; Russell, 2019). On the other hand, this is part of an even broader debate concerning the question to what extent we as a society can control the innovation process, and seeing to it that it really serves some relevant, long-term, human and societal interests. This in turn depends on the extent to which technological processes are responsive to values and principles reflectively endorsed through open and democratic debates among experts and other relevant stakeholders. Famously inspired by (Collingridge, 1980)’ book *The Social Control of Technology*, these studies have now been developed under the name of Responsible Innovation (Stilgoe et al., 2013).

The theory of “meaningful human control” (MHC) presented and discussed in this paper lies somewhere in between these two approaches to control. On the one hand, like the Responsible Innovation program from which it takes inspiration, MHC describes a control philosophy, not an operational control theory. It defines and prescribes the conditions for a relationship between controlling agents and controlled system that preserves moral responsibility and clear human accountability, even in the absence of any specific form of operational control from a human operator. On the other hand, when applied to specific technical systems, such as automated driving systems, MHC also has the ambition to be translated and operationalised in terms that can be used by engineers, designers, policymakers and others to define the tasks, roles, responsibilities, abilities of different operators and human agents in the design control regulation use chain.

When referring to control in this paper, we refer to control from a sociotechnical perspective of influence over a system. When referring to a ‘system’, from a generic philosophical point of view, this can be any system, while in this paper the considered system is that of an ‘Automated Driving System’ unless otherwise explicitly mentioned. By that, we mean to indicate the broader sociotechnical system surrounding the autonomous driving enterprise in its entirety. This is made of humans, societal components, e.g. drivers and policy makers, as well institutional, e.g. traffic regulations, and technical ones, e.g. the specific solutions and artifacts.^1^

Santoni de Sio & Van Den Hoven (2018)’s account of MHC aims to provide both a solid theoretical framework (grounded on a philosophical theory of responsibility and control (Fischer & Ravizza, 1998)) and an applied, value-sensitive, design perspective on control. Their approach proposes that, in order for intelligent systems to be meaningfully under control of human agents, two main conditions have to be satisfied, called "tracking" and "tracing". The first criterion, tracking, focuses on the nature of the relationship between human controllers and controlled intelligent systems. The fulfilment of the tracking criterion depends on the degree to which a system can “track” the intentions or the “reasons” of its designated controller(s). A higher tracking value is achieved by improving the capacity of a system to seamlessly respond to its controller(s)’ reasons. We can immediately see how this criterion embodies MHC’s innovative potential. Whereas classic control theories in engineering put the accent on the quality and quantity of the causal, operational relation between a controller and a controlled system, MHC theory proposes to base control not –just, or mainly– on a causal relationship, but on a more abstract coordination. Namely, on the degree to which the behaviour of a system is aligned to, and capable to covary with, the moral reasons, the intentions, scopes and goals of its controller(s). The implication of the tracking criterion, which is defined as such in ethics and philosophical literature, is that it allows taking into account among controllers of a system even agents that are not directly, i.e. operationally, in control. The rationale behind becomes clearer if we consider that this theory of control is designed to grant a reliable, reliably retrievable, connection between designated human controllers and autonomous (even fully autonomous) machines, which by definition do not require any form of operational control. Also, the theory specifies that in considering the intentions of the controllers, we should consider them in their moral relevance, i.e. in their being relevant for a moral evaluation of the system’s behaviour. This is the case because meaningful human control theory, as said, is designed to respond to the need of preserving human moral responsibility in those situations where “gaps” would otherwise occur. More generally, as explained above, this depends on the ambition of the theory of MHC to connect the concept of control over AI systems to Collingridge’s concept of “social control of technology” and the Responsible Innovation literature.

Whereas the tracking criterion mainly focuses on the quality of the relation between controllers and controlled systems, the tracing criterion concerns more closely the capacities of human controller(s) and the nature of their involvement in the chain of control. This criterion prescribes the presence of at least one, ideally more, persons in the system design history or use context who can (i) appreciate the capabilities of the system and (ii) their own role as target(s) of potential moral consequences for the system’s behaviour. Such person(s) would be suitable, to the extent they fulfil the criterion, to be designated as controllers, and consequently to bear responsibility for the consequences of the actions of the system they control. To further clarify, requirement (i) concerns the quality of the physical and cognitive capacities of the controller in relation to the controlling tasks. A controller is more meaningfully in control of a system the more they possess practical skills (know-how) and theoretical knowledge (know-that) of its functioning. Correspondingly, the system should be designed to match the technical and psychological capabilities of the users.

To be sure, the application of the theory is very context-dependent. Whereas, to avoid responsibility gaps of various kinds, a system should respond to some relevant reasons of some relevant agents (tracking), the theory leaves open who these agents and their reasons may be. Also, while stating that there must be at least one agent that possesses both sufficient technical expertise and moral awareness (tracing), the theory leaves open whether these agents are the same fulfilling the tracking condition. Moreover, the extent to which any human agent fulfils the two criteria of tracking and tracing determines the degree of their involvement in controlling the behaviour of a given system, and hence their suitability as potential bearers of different forms of responsibility. Multiple agents may be, according to MHC theory, deemed in control of a system by fulfilling different criteria to different extent. Determining which degree of MHC an agent should exercise to be a suitable target for moral responsibility, and the exact amount and nature of this responsibility, is beyond the scopes of the theory. That would indeed depend on further philosophical, cultural and social aspects. Rather, the theory means to provide with a set of criteria (tracking and tracing, together with their sub-conditions) that are relevant to assess control and responsibility in high autonomy scenarios, where the operator’s role is no longer the most prominent, nor the most important.

## Operationalising the “Tracking” and “Tracing” Conditions for Meaningful Human Control

MHC is a philosophical theory that contains abstract, inherently normative components. This is the case as the theory is aimed to address ethical, legal and societal impacts of intelligent, autonomous technology, and to help addressing various forms of the responsibility gap. However, to translate the normative requirements of MHC into workable design solutions, that theory needs “handles” from behavioural science and engineering design to (i) investigate how and to what extent humans can comply with those requirements and (ii) realise systems that maximise the possibility of such compliance. In other words, the different theoretical and normative requirements need to be operationalized to make them as close as possible to a scientifically quantifiable and measurable set of criteria. This operationalisation is non-trivial, since ethical notions such as that of responsibility, or philosophical ones like “reason-responsiveness”, are not meant to be quantified, but they are destined to maintain a qualitative element. One of the instruments that philosophy has been using for a long time is that of conceptual analysis and clarification, often mediated by the formulation of schemes and taxonomies. By relying on recent literature in philosophy, behavioural psychology, and traffic engineering, this paper shows how this gap between theory and practice can be reduced by operationalizing MHC into a more workable, applicable theory of control. By spelling out and operationalizing the notions of tracking and tracing in relation to ADS, we show how these notions can eventually address the major problem from which the theory of MHC originated: the responsibility gap.

### Operationalizing “Tracking”

The condition called “tracking” requires a reliable alignment between human controllers’ (moral) reasons and the behaviour of the controlled system. We consider “(moral) reasons” as a category that includes all intentions, goals and plans, potentially morally loaded, that an agent entertains. The tracking requirement does not provide any clue on how to identify a set of relevant agents for a given situation, and tell apart the different reasons that can (or should) influence a system’s behaviour. In other words, we don’t have a way to find out, or even make clear, which agents and which reasons a system is or should be responsive to. This is for the most part a societal process of normative decision making that cannot be—and should not be—standardized once and for all. However, the process of identifying relevant agents and relevant reasons for a certain behaviour of a system, can be aided and clarified by providing some extra criteria and dimensions. Mecacci and Santoni de Sio (2020) used behavioural psychology (Michon, 1985) and philosophy of action and agency (Anscombe, 1957; Bratman, 1987) to develop a “proximity scale of reasons”, a conceptual tool that provides a criterion—proximity—to correlate (a) the different relevant human agents (individuals as well as supra-individual agencies) and (b) the different intentions and reasons the system does, or should, respond to, to (c) the behaviour of the observed system. The scale provides therefore a (preliminary) model to understand the complex reciprocal relations among multiple potential controllers and their influence towards the controlled system’s behaviour. It also provides insights on how to design systems to be (more) responsive to chosen agents.

**Fig. 2:**
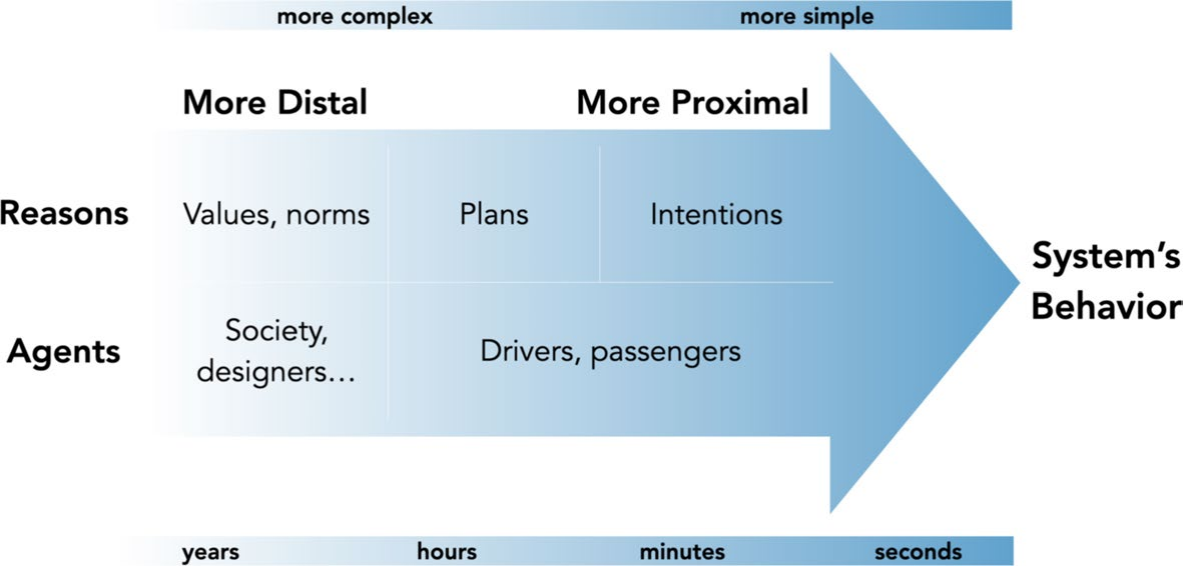
Proximity scale of reasons (from Mecacci & Santoni de Sio, 2020)

The proximity scale in Fig. 2 shows how different agents, together with their relative values, goals and intentions, are positioned according to how “proximal” they are to a certain considered behaviour of the system. On the extreme left, reasons and agents are further removed in time and space from the system’s behaviour. They are also more complex and encompassing e.g. overarching goals rather than simple intentions. The proximity scale has been successively integrated into a broader taxonomy to bring it one step closer to a concrete operationalization in the context of ADS. We will see in Sect. 5 what the taxonomy entails in terms of traffic modeling.

### Operationalizing “Tracing”

The tracing condition is markedly normative, in the sense that it sets a number of explicit requirements on the designated human controller(s). The idea is that such controller(s) can be meaningfully deemed in control only inasmuch as they are cognitively, physically and morally capable to perform their assigned tasks and fulfil their obligations. However, these capacities are abstractly defined in the sense that it is harder to evaluate, let alone quantify, the degree to which a controller possesses them. In order to provide some quantitative ground to an otherwise purely qualitative assessment of these capacities and requirements, (Calvert et al., 2019) devised what they called an “evaluation cascade” that would base a subjective evaluation of certain aspects on a 6-point likert scale. Likert scales are common in psychology to poll *perceived* amounts. The evaluation cascade table is of course a compressed concept version, one that can be expanded, however, and refined to better comply with potentially different needs of its users, typically policy makers and engineers, but not necessarily so. Table 1 shows four of the main aspects that need to be considered in evaluating the fulfilment of the tracing condition: the exertion of operational control, the involvement of a human agent, the ability of that agent to understand and use the system, and the ability of the agent to understand their moral responsibility over the system. The first two aspects, A and B, focus mainly on the presence of operational control and the involvement of humans. Without vehicle operation, there can be no control, and without identifying human involvement MHC can by definition not be present. Aspects C and D address the explicit conditions for tracing. For each aspect, each potential agent is given a score along the 6-point likert scale that reflects the degree of such aspect for the agent. It is important to remember that exertion of MHC is not binary and that we must consider the extent to which control is exerted. The critical score for each aspect (A, B, C, D) is the highest score from all human agents, because if one agent can achieve perfect performance for that aspect, other aspects are less important as control may already be guaranteed. However, each aspect needs to be considered as part of assessing overall MHC from the point of traceability. Therefore, the aspects are set out in a cascade in which the score from the proceeding aspect and the current aspects are compared to determine the critical score for that aspect. In this case, we take the minimum score from that aspect and the previous aspect to determine the current critical score up to that point. Therefore, the critical scores for aspects B, C and D, are influenced by all the aspects that have preceded them. The final score of the system regarding the fulfilment of the tracing condition is the critical score produced by D.

**Table 1:**
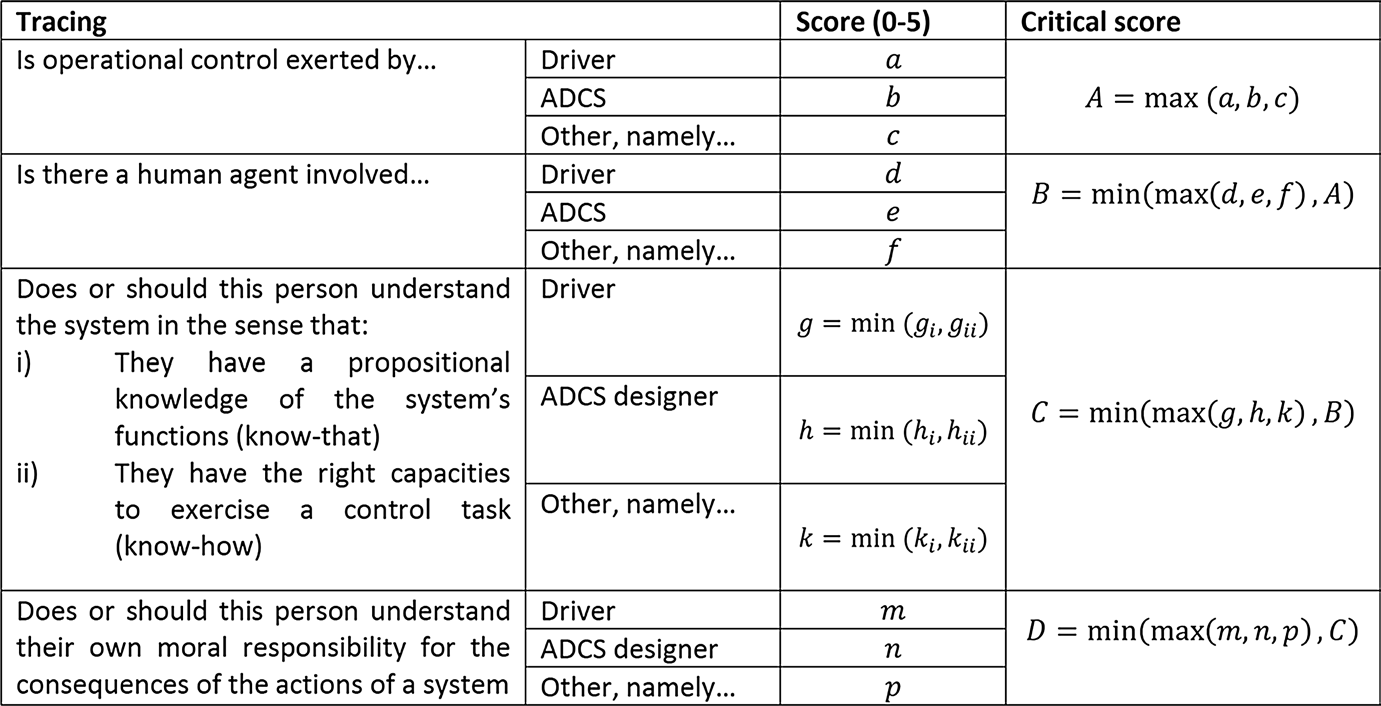
Evaluation cascade table for the tracing criterion (Calvert et al., 2019)

## The Behavioural and Driver Perspective

Both the tracking and tracing criterion for meaningful human control require an appraisal of the reasons and capabilities of human actors involved in the design, control, use of ADS. Among human actors, drivers remain relevant in the use of ADS. Human drivers may be considered to be fairly competent at controlling their vehicle, insofar as they have been trained to do so through mandatory driver training schools and experience. Ensuring drivers’ competence is one necessary condition to achieve meaningful human control, insofar as this contributes to the alignment of ability, control and (moral) responsibility (Flemisch et al., 2012). In the absence of such an alignment, the driver may find themselves in what has been called the “moral crumple zone” (Elish, 2019), a space in which they are blamed and possibly held legally liable for something they did not have the ability to control. When a human is driving a vehicle, (s)he uses their skills, applies rules (s)he learnt, and adapts to an unknown situation using their knowledge from other (similar) situations (Rasmussen, 1983). However, with the introduction of automated driving systems (ADS), several driver tasks are being taken over from human drivers to be executed by the ADS, and new monitoring and intervention tasks are introduced. From a MHC perspective, this prompts the question, how to keep human drivers’ capabilities aligned with the functioning of ADS. At the lower levels of automation (SAE levels 1 and 2) an ADS only carries out the basic control tasks, namely accelerating, braking, and steering, leaving the more complex tactical and strategic tasks to the human, such as overtaking, merging, and for instance driving along a roundabout. Moreover, the system requires the human driver to monitor the automated system for possibly extended periods of time, introducing novel tasks which humans are notably poor at (cf. Mackworth, 1948). This is where the so-called “unsafe valley of automation” begins (Flemisch et al., 2017). At a higher level of automation an operator controlling the vehicle remotely might ultimately have control over the vehicle instead of the driver, which might alleviate the driver from some untaught tasks, but could possibly also introduce increasingly complex novel tasks. In this section we first study MHC from a behavioral and driver perspective in terms of task classification, driver training, and human machine interfaces (HMIs). Next, we study how consideration of personal characteristics in terms of Big Five personality traits (Openness, Conscientiousness, Extraversion, Agreeableness, and Neuroticism; Norman, 1964) can contribute to realising the tracking condition for MHC.

### Driving Task Classification, Driver Training, and Human Machine Interfaces

The introduction of novel tasks may result in a mismatch between supply and demand of behavioural capabilities of the human driver. In order to inventory this potential mismatch, Heikoop et al., 2019 developed a framework of the skill-, rule-, and knowledge-based behaviours (cf. Rasmussen, 1983) with the SAE levels of automation (SAE International, 2018, see Sect. 1 above). Heikhoop et al.’s framework was used to quantify the amount of tasks needed, left, and introduced at the various levels of an ADS. The resulting framework identified a critical gap between the supply and demand especially revolving around SAE level 3, where the ADS is capable of performing the entire dynamic driving task (DDT), given its operational design domain (ODD), but the human is still considered the fall-back in case of emergency (SAE International, 2018).

Heikoop et al. (2020c) report the result of a focus group discussion with 11 Dutch driver examiners. The goal of the focus group was to further the implementation of the MHC principles into practice through the opinions and experiences of field experts. One main finding of the driver examiners was that they consider the current market introduction of ADAS improper, because, as it stands, no one is or feels responsible for ensuring proper driver understanding of the ADAS functionalities within consumer vehicles, which is key for safe and meaningful control over such a vehicle (see also Beedham (2020) for recent news on this topic). Moreover, it was discussed whether driver training with ADS should at all exist, as some argued the learnability of these types of tasks is moot, and at this point responsibility should not lie with the driver. Some would prefer learning through experience instead, but this would raise the question of who would or should be responsible for the behaviour of an ADS when the driver is still not experienced enough? This further complicates addressing the responsibility gap in relation to MHC over ADS. This safety—critical situation was further supported by the notion of automation surprise (cf. Bainbridge, 1983), which the focus group explicitly considered to be a serious issue related to the introduction of ADAS. To facilitate safe usage of ADAS or ADS, the focus group urged to have ADAS intuitive, easy, and fun, and (perhaps most importantly) to not have drivers be required to monitor an ADS as a key task during driving, nor to have it be a part of future driver training as that was considered not viable, since it requires extensive training and certain personal characteristics not possesses by everybody (cf. e.g., Young et al., 2007). The notion of personal characteristics is an important one, as it is also recently shown that different personalities have different ways of (appropriate) training (see e.g., Zahabi et al., 2021), and thus investigating individualization of training with ADS appears key.

In order to develop tailor-made or tailor-taught (training for) ADS, Human–Machine interfaces (HMIs) are considered to be promising, as they could allow real-time, flexible, and personalised information and/or warnings, and are utilizable to engage the driver in the driving task, contributing to realising the tracing condition of MHC. Thus, assumedly playing an important role in safely driving an ADS, the scientific domain could do with an overview of the current state-of-the-art regarding HMIs in ADS, as that was until now still lacking. A total of 340 papers were categorised within four main categories, and 21 subcategories, and a large amount of statistics in terms of methods and materials used in the papers were reported (Gürses, 2020). Comfortingly, it was found that research involving HMIs in ADS appear to stay able to keep the pace of the rapidly developing technology behind ADS. Furthermore, HMIs appear to be predominantly researched to provide visual feedback, especially for either SAE level 0 (i.e., manual driving) or 3 (conditional automation). Since the driving task is primarily a visual task, this is not surprising; also (combinations with) auditory and haptic feedback are being studied—with varying results. Interestingly, it appears that much attention is still paid to informing the driver through an HMI, already at the entry level (SAE level 0). If, as suggested above, HMIs are currently not yet able to appropriately inform, let alone warn or engage the driver, to what extent can we consider a driver to be under MHC over his/her ADS? This suggests that the development of HMIs is still in its relative infancy (cf. Carsten & Martens, 2019), causing concern about the current state of both ADS and HMIs in relation to MHC. Instead of aiming for HMIs to advance in pace with AD(A)S technology, we should also consider how HMIs (and therewith ADS altogether) could function best for any given individual, as some likely need more guidance than others, as mentioned above.

### Personality and the Tracking Condition for Meaningful Human Control

As an initial step towards a full-fledged personalised ADS, it is helpful to start from the distinctions between the Big Five personality traits: Openness, Conscientiousness, Extraversion, Agreeableness, and Neuroticism (Norman, 1964). A preliminary research (103 participants) towards individual differences based on these Big Five traits (using an algorithm developed to equally and fairly distribute participants over the five traits (Heikoop et al., 2019) resulted in some indications towards, among others, the confirmation of the stereotypical rich, older male being a tech-lover, and that extraverted people tend to report having more experience with ADS compared to other personalities. An online questionnaire amongst 120 participants towards the relationship between personalities and trust in ADS showed a moderate correlation between highly conscientious and nominally neurotic females, both scoring high on trust towards ADS, while on the whole, ADS appeared to be trusted equally, regardless of the person’s personality (Heikoop et al., 2020a, 2020b, 2020c).

As followups on those studies, some of the authors of this paper made preparations for a large-scale simulator study towards the effects of personality on driving with ADS in which participants divided over the five traits would take part in one study investigating the effects on workload during a take-over request (TOR), and on the response to an auditory TOR in terms of take-over quality. Due to the COVID-19 pandemic, the preparations were developed and executed as two N = 1-studies designed to validate relevant variables within the experimental design by the lead researchers themselves. The main findings of these studies were that the standard deviation of normal-to-normal peaks (SDNN) of the heart rate is a useful measure for measuring workload variations during a TOR, that a learning curve exists for lane keeping based upon 81 iterations, and that the level of urgency of a TOR can be distinguishably tested (Ebbers, 2020; Marfoglia, 2020). The findings provide a solid basis for conducting a future large scale simulator with a large number of participants as soon as restrictions due to the COVID-19 pandemic allow.

From a human factors and driver training point of view, meaningful human control is a long way from reaching maturity. The apparent mismatch between what a driver is expected to and what (s)he is capable of doing within an ADS has by now become an obvious statement, yet still a persistent problem to overcome. Whether the way forward towards meaningful human control is through individual, personalised ADS is still open for debate; several differences between personalities and their effect on (driving with) ADS have been elicited. The research reported in this paper aimed to make an initial step towards empirical verification of the tracking condition, that is, in this case, the connection between the driver’s reasons and the ADS behaviour. In spite of limitations in the research set up to COVID 19, empirical verification appears feasible. Our preliminary results showed that drivers’ intentions do not necessarily add up to their actual behaviour, which could prove to be an obstacle for fulfilling tracking with ADS (see Struik (2021) for more details). Simply put: if human drivers do not fulfil the tracking condition, why should ADS? It can therefore be concluded that more research is needed, that we seriously ought to try and train people to appropriately interact with their increasingly automated automobiles (cf. e.g., Boelhouwer et al., 2020; Manser et al., 2019; Merriman et al., 2021), and that (adhering to the conditions of) MHC promises to be a fruitful way forward towards safe control over automated driving systems.

## The Engineering Approach

If MHC is to have any tangible practical relevance beyond high level discussions, it needs to be able to connect to concrete processes involving the development of ADS’s, but also consider human capabilities and moral understanding as we saw in the previous sections. Primarily, we envisage that MHC can be utilised on two fronts: for evaluation of current and proposed systems; and for the design of new systems. As an instrument for evaluation of ADS performance, with the explicit consideration of human reasons, MHC can be applied to flag shortcomings in system design that overlook human responsibility. For design, the objective would be to solidly integrate MHC conditions into design decisions to enable the system to be designed to meet human moral responsibility set out in the concept of MHC. Operationalisation of MHC is required to allow MHC to be used for these purposes. This comes with its own challenges, as were highlighted in Sect. 3. In the remainder of this section, we will consider our approach to operationalisation of MHC and some of these recent examples to implement them.

### Operationalisation of MHC for Road Traffic Automated Driving Systems

In general, evaluation and design can be approached from a qualitative or quantitative perceptive. A qualitative approach allows indicative evaluation of performance to take place and does not require an as mathematical quantification of MHC and therefore can be more easily constructed using the general conditions of tracking and tracing of MHC in a descriptive manner. On the other hand, a quantitative approach allows fine tuning of a system and a greater degree of design flexibility. To achieve a quantification of MHC, the concept has to be translated from philosophical and behavioural terms into physical control formulations for mathematical description.

**Fig. 3:**
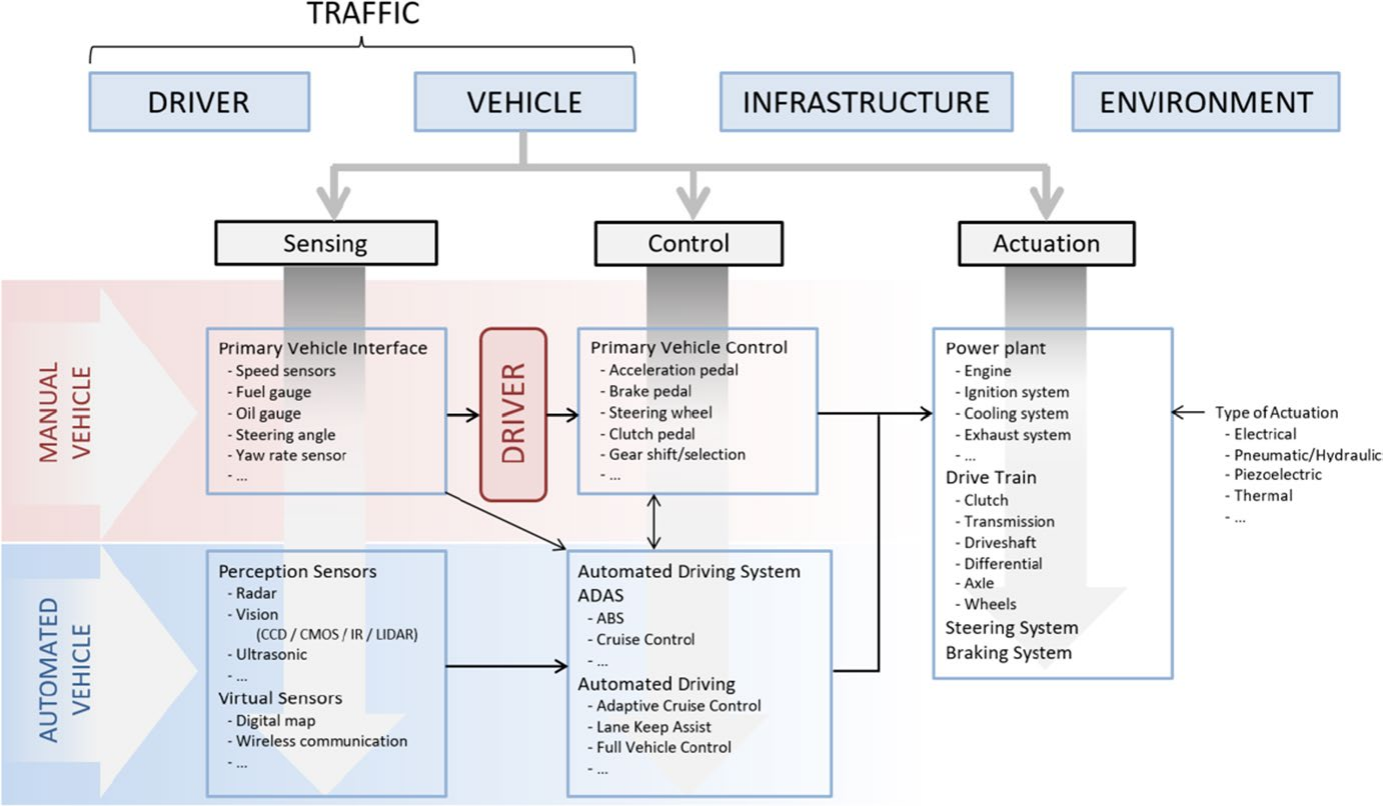
Vehicle core components for automated driving (Calvert et al, 2020)

Quantification of MHC requires a deeper understanding and interconnected definition of the underlying dynamic processes, which equally aids the construction of qualitative approaches. To be able to achieve this, a taxonomy of the key core components of the systems must be constructed. Based on state-of-the-art literature from many sources, including (Amditis et al., 2012; Chandrasiri et al. (2016); Farah et al., 2018; Ibañez-Guzman et al., 2012; Körber et al., 2015; McKnight & Adams, 1970; Sanchez et al., 2016; Theologus & Fleishman (1971)), Calvert et al. (2020a, 2020b) constructed such a taxonomy identifying the core components for the Vehicle, Driver, Infrastructure and Environment systems An excerpt of the taxonomy for the Vehicle category is shown below in Fig. 3.

Let us first focus on the approach for qualitative evaluation before turning our attention to the latter.

As can be derived from the descriptions of tracing and tracking, these are conditional processes that are suitable for a Markov style approach. The ‘Tracing’ condition, for example, states that control is exerted by one or more individuals, which could occur at different points in *time* on different aspects of the system (e.g. design, monitoring, physical control). Also, once identified, these persons need to be considered for their system knowledge, and their own capabilities, which focuses on a *conditional role*. Furthermore, their understanding that their own actions have moral consequences also follows this, which equates to a *causal relationship*. These aspects of time dependency, conditional roles and causality, are well suited for a what-if-style approach. The cascade approach suggested in Table 1, Sect. 3 starts by evaluating higher level components of the tracing conditions, e.g. is there a human involved and where, then moves (or cascades down) to lower level components, e.g. agents knowledge of the system, and evaluates them, and so on (see Table 1). In the end, the final score is a conditional and causal intersection of the components of the tracing condition and gives a qualitative evaluation score of the extent to which the tracing conditions is met. In the same paper, this is demonstrated with a case on a dual mode (human-ADCS) controlled automated vehicle. This initial application of the approach showed that it is relatively easy to assess if operational control is asserted and by whom, with critical scores for the ADCS being 5 and 4 out of 5 respectively for these two aspects. However, for the aspects concerning a person’s far-reaching understanding of a system's effects and function in practice and of their own role as being morally responsible, we see much lower scores of 2 out of 5 for both of these aspects. This results in a final score of 2/5 for this case and clearly highlights the challenges that automated vehicle designers and developers have to effectively incorporate meaningful human control into automated vehicle system design.

**Fig. 4:**
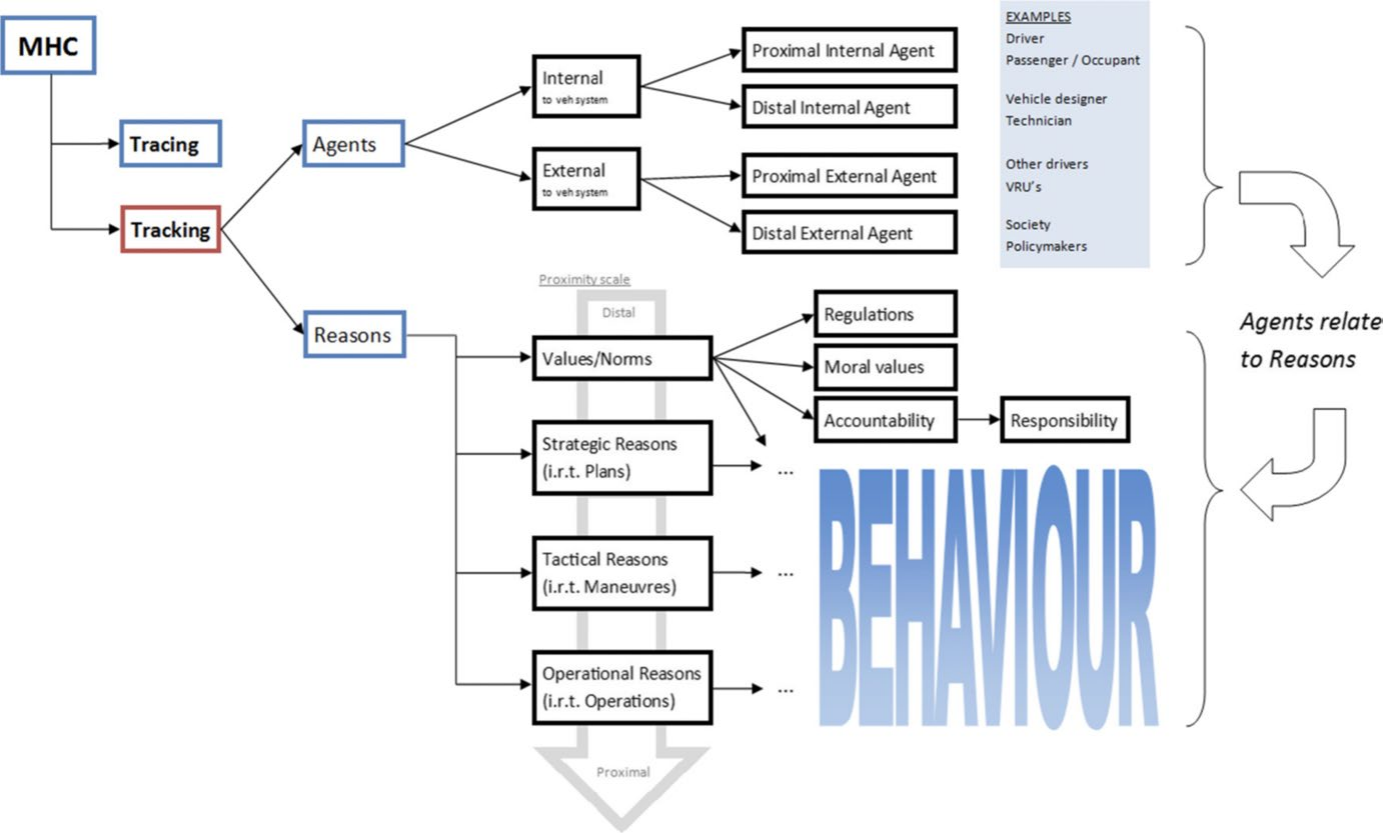
Relational framework for the operationalisation of Tracking (Calvert & Mecacci, 2020)]

For quantitative approaches, (Calvert & Mecacci, 2020) start with the aforementioned taxonomy of components. The two main conditions (tracking and tracing) are conceptually translated from philosophical and ethics descriptions into a framework of solid technical and cognitive connections by making use of the components. The outcome of this for the tracking condition is demonstrated in Fig. 4.

From the conceptual framework, mathematical formulations of the various different interactions can be derived, making use of a combination of behavioural, cognitive and engineering literature. As many connections are insufficiently investigated or rely heavily on constructs, suitable and well-founded assumptions must be made to allow formulations to be constructed. The influence of moral responsibility (in tracking), or the learning capabilities of drivers (in tracing) are such examples where current knowledge is present, but insufficiently from a generic point of view to be able to generalise equations based on empirical evidence without the use of assumptions. Furthermore, many connections cannot even be feasibly assumed and must therefore remain at a conceptual level without immediate mathematical description for the time being. A good example is the dynamical role of societal values, and their influence on acceptable risk and danger for example. To describe such a variable is a massive undertaking in itself and is therefore not feasible in the short term for a demonstration of MHC operationalisation. The eventual extent of operationalisation into mathematical formulations was limited in Calvert and Mecacci (2020) to the variables that allowed a specific use case to be considered. This use case focussed on a highly automated vehicle on an urban road that gradually learns how to overtake a cyclist while adhering and optimising towards human reasons that are formulated in terms of tracking and tracing with a focus on traffic safety and a timely arrival at the vehicle’s destination. The case offered insights into the dynamic influences of different MHC influencing components and demonstrated that MHC can be quantitatively applied as a design and optimisation approach to let automated systems explicitly consider MHC.

### Current Progress and Outlook for Engineering

The aforementioned qualitative and quantitative descriptions of MHC operationalisation act as a demonstration that MHC can indeed be used for evaluation and design of automated systems. This opens the door to unleash the potential that MHC has to positively influence the development and application of the systems, which also includes regulatory, policy and legal considerations. While the feasibility has been proven, this is only the start of the process of more expansive operationalisation of MHC in applications. Researchers from the various involved domains of philosophy, ethics, cognitive science, psychology and control and applied engineering need to come together to further connect the different components and investigate how they can be further quantitatively described based on strong empirical evidence. Furthermore, the onus is very much on regulators and OEM developers alike to choose to implement policy and apply MHC for the development and evaluation of automated driving systems to ensure that human ability is properly considered and that systems adhere to human reasons in a proper fashion. It is not enough to merely state they should without actually involving the underlying conditions and premise of MHC to be applied.

## Synthesis, Discussion and Future Research Directions

The main scientific contribution of this paper is a framework to realise Meaningful Human Control in the design, development and deployment of Automated Driving Systems. The framework connects abstract and normative principles with realistic and complex application scenarios of automated driving systems. A fundamental assumption—in line with general theory on Meaningful Human Control (Santoni de Sio & van Hoven, 2018)—is that humans, not hardware and software and their algorithms should remain ultimately in control of, and thus morally responsible for, the potentially dangerous operation of automated driving systems. In short, we assume that an Automated Driving System is under Meaningful Human Control if (1) it behaves in accordance with the relevant reasons of the relevant human actors (tracking) and (2) any possibly dangerous event can be traced back to human actor(s) equipped with the relevant technical, psychological and moral capabilities (tracing).

We have given indications in this paper that Meaningful Human Control can be successfully operationalised for automated driving systems using a multidisciplinary approach combining moral philosophy with traffic engineering and behavioural sciences. We defined a conceptual methodology to operationalise the tracking condition in relation to ADS. We developed a method that enables to systematically identify responsibility gaps in a driving system.

The decomposition into core components was used to identify the task demands of the human actors involved and skills to fulfil these tasks in a responsible way. A comprehensive review of the literature shows that there is a huge gap in the understanding of the interaction between Automated Driving Systems and relevant human actors at the strategic driving tasks which typically are tasks involving value sensitive goals and intentions. A steadily increasing body of literature on the interaction between Automated Driving Systems and driver concerning the tactical and operational driving tasks suggest a mismatch between driver capabilities and task demand, leading to risks that currently may be overlooked, undervalued or implicitly societally accepted.

The decomposition into core components was used also to study the virtual operation of Automated Driver in traffic flow simulation. Furthermore, the formulation of the framework and development and application of the research methods were carefully chosen and turned to be a powerful and effective channel of communication, allowing stakeholders to translate project findings into their own fields such as the Horizon 2020 Commission Expert Group to advise on specific ethical issues, UN ECE Harmonization of Vehicle Regulations and the Dutch Legislation for Experiments with Self-Driving Vehicles.

In order to develop, detail and apply the framework of Meaningful Human Control of Automated Driving Systems, the following important limitations were assumed.

- We consider the right to safety and human responsibility as the main values at stake, while evidently the ethics of automated driving involves a wider set of values such security, fairness, privacy (H2020 EC Expert group, 2020). For a full consideration of ethics of automated driving systems, a wider scope of the values at stake is needed (H2020 EC Expert group, 2020).
- The applications of the framework mostly applied to desk research and simulations. In addition, the driving simulator experiments suffered from restrictions due to the 2020 Covid 19 pandemic. Feedback from real-world pilots and field test was limited, but will have to be taken into consideration to get a comprehensive understanding of Meaningful Human Control of Automated Driving Systems.
- The institutional embedding of the framework has not been addressed specifically. Although the research has assumed EU vehicle type approval procedures and legal conditions, the framework and its core component are also applicable other conditions. In the US, Waymo (2020) addresses values and core components such as safety, responsibility and driving scenarios in their safety report.

Suggested directions for future research are based on the elaboration of meaningful human control as a cornerstone to value based responsible R&D into automated driving.

- Further philosophical and empirical research into the connection between better responsibility mechanisms and road safety
- Extension of the MHC-ADS framework toward other relevant values such as privacy and fairness EC (2020).
- Behavioural research into the alignment of intentions (tracking) and capabilities (tracing) between the driver and automated driving systems. This research should in particular be aimed at the strategic and tactical driving tasks and establish conditions to enhance user acceptance and reduce accident risk.
- Behavioural and technical training and testing requirements and programs for both driver and automated driving systems.
- Develop and assess value sensitive driving strategies and traffic flow simulations, including transitions of control and enabling verification of tracking and tracing conditions.
- Application of the MHC-ADS framework to additional automated driving systems such as shared (L2/L3), remote supervised (L4) and/or shared space (L2/3/4) automation.
- Empirical studies using a driving simulator, pilot project and field trials into perceived alignment of intentions (tracking) and responsibility gaps (tracing).
- Research into the translation of the principle of MHC into institutions, policy and governance.

## Conclusion

High expectations rest on automated driving systems to improve safety, comfort of driving and improving traffic flow efficiency. At the same time the development of AI-based intelligent systems raises strong ethical concerns for safety and human responsibility. The approach of Meaningful Human Control over automated Driving Systems assumes that humans should ultimately remain in control of and be morally responsible for the safe operation of the automated driving systems. The elaboration and application of Meaningful Human Control in this paper is based on the values of safety and human responsibility and follows the alignment of human reasons (tracking) and capabilities (tracing) and the automated driving system. This paper not only puts forward the scientific basis from moral philosophy, but also a translation into scientifically grounded methods from behavioural science and traffic engineering. Further research is proposed into a broader set of values, behavioural and traffic flow research, additional use cases and empirical studies.

## Data Availability

Not applicable.

## Footnotes

1 For more details on the notion of “system” we are adopting, we refer the reader to Mecacci & Santoni de Sio (2020), in particular the section “Meaningful Human Control, tracking and the role of reasons”. At that point and several others in that paper, the philosophical nature of the “system” that is meant to be under MHC is discussed.

## References

[CR1] Anscombe, G. E. M. (1957). Intention. Harvard University Press.

[CR2] Amditis A, Panagiotis L, Evangelia P (2012). Sensing and actuation in intelligent vehicles. Handbook of Intelligent Vehicles.

[CR3] Bainbridge L (1983). Ironies of automation. Automatica.

[CR4] Beedham, M. (2020, December 18). “Autonomous vehicle makers should be held responsible for accidents, says Law Commission”, SHIFT. https://thenextweb.com/shift/2020/12/18/autonomous-vehicle-makers-should-be-held-responsible-for-accidents-says-law-commission/. Accessed 18 Dec 2020

[CR227] Boelhouwer, A., van den Beukel, A. P., van der Voort, M. C., & Martens, M. H. (2019). Should I take over? Does system knowledge help drivers in making take-over decisions while driving a partially automated car? *Transportation research part F: traffic psychology and behaviour*, *60*, 669–684.

[CR5] Boelhouwer A, van den Beukel AP, van der Voort MC, Verwey WB, Martens M (2020). Supporting drivers of partially automated cars through an adaptive digital in-car tutor. Information.

[CR6] Bonnefon J-F, Černy D, Danaher J, Devillier N, Johansson V, Kovacikova T, Martens M, Mladenovič M, Palade P, Reed N, Santoni de Sio F, Tsinorema S, Wachter S, Zawieska K (2020). Horizon 2020 Commission Expert Group to advise on specific ethical issues raised by driverless mobility (E03659) Ethics of Connected and Automated Vehicles: recommendations on road safety, privacy, fairness, explainability and responsibility.

[CR8] Bostrom N (2014). Superintelligence: Paths, Dangers Strategies.

[CR235] Bratman, M. (1987). Intention, Plans, and Practical Reason. Cambridge: Cambridge, MA: Harvard University Press.

[CR221] Calo, R. (2015). Robotics and the Lessons of Cyberlaw. *California Law Review*, *103*(3), 513–563. 10.2139/ssrn.2402972

[CR10] Calvert, S. C., Mecacci, G., van Arem, B., de Sio, F. S., Heikoop, D. D., & Hagenzieker, M. (2019). Gaps in the control of automated vehicles on roads. IEEE intelligent transportation systems magazine.

[CR11] Calvert SC, Heikoop DD, Mecacci G, van Arem B (2020). A human centric framework for the analysis of automated driving systems based on meaningful human control. Theoretical Issues in Ergonomics Science.

[CR12] Calvert SC, Mecacci G (2020). A conceptual control system description of cooperative and automated driving in mixed urban traffic with meaningful human control for design and evaluation. IEEE Open Journal of Intelligent Transportation Systems.

[CR13] Carsten O, Martens MH (2019). How can humans understand their automated cars? HMI principles, problems and solutions. Cognition, Technology, & Work.

[CR14] Chandrasiri NP, Nawa K, Ishii A (2016). Driving skill classification in curve driving scenes using machine learning. Journal of Modern Transportation.

[CR212] Coeckelbergh, M. (2020). Artificial Intelligence, Responsibility Attribution, and a Relational Justification of Explainability. *Science and Engineering Ethics*, *26*(4), 2051–2068. 10.1007/s11948-019-00146-8 10.1007/s11948-019-00146-8 PMC7417397 31650511

[CR230] Collingridge, D. (1980). The Social Control of Technology. Frances Printers.

[CR219] Danaher, J. (2016). Robots, law and the retribution gap. *Ethics and Information Technology**18*(4), 299–309. 10.1007/S10676-016-9403-3

[CR226] Delvaux, M. (2017). Report with recommendations to the Commission on Civil Law Rules on Robotics (A8-0005/2017).

[CR224] Doran, D., Schulz, S., & Besold, T. R. (2017). What Does Explainable AI Really Mean? A New Conceptualization of Perspectives. CEUR Workshop Proceedings, 2071.

[CR15] Ebbers, T. (2020). Does personality affect responses to auditory take-over requests? Validating a simulator experiment setup through a N=1-study. MSc thesis. Delft University of Technology, Delft, The Netherlands. https://repository.tudelft.nl/islandora/object/uuid:14eb3124-d344-48de-9315-5527e8468e58. Accessed 18 Dec 2020.

[CR213] Edwards, L., & Veale, M. (2017). Slave to the Algorithm? Why a Right to Explanationn is Probably Not the Remedy You are Looking for. *Duke Law and Technology Review*, *16*(1), 1–65. 10.2139/ssrn.2972855.

[CR16] Elish MC (2019). Moral crumple zones: cautionary tales in human-robot interaction. Engaging Science, Technology, and Society.

[CR228] Ekelhof, M. (2019). Moving beyond semantics on autonomous weapons: Meaningful human control in operation. *Global Policy*, *10*(3), 343–348

[CR17] Fagnant DJ, Kockelman K (2015). Preparing a nation for autonomous vehicles: opportunities, barriers and policy recommendations. Transportation Research Part A.

[CR18] Farah H, Erkens SMJG, Alkim T, van Arem B (2018). Infrastructure for Automated and Connected Driving: State of the Art and Future Research Directions. Road Vehicle Automation 4.

[CR231] Fischer, J. M., & Ravizza, M. (1998). Responsibility and control: A theory of moral responsibility. Cambridge university press. Chicago.

[CR19] Fleiter J, Watson B, Group Research Co-ordination Advisory (2005). The speed paradox: the misalignment between driver attitudes and speeding behaviour. Australasian Road Safety Research, Policing & Education Conference.

[CR20] Flemisch F (2017). Uncanny and Unsafe Valley of Assistance and Automation: First Sketch and Application to Vehicle Automation. Advances in Ergonomic Design of Systems Products and Processes.

[CR21] Flemisch F, Heesen M, Hesse T, Kelsch J, Schieben A, Beller J (2012). Towards a dynamic balance between humans and automation: Authority, ability, responsibility and control in shared and cooperative control situations. Cognition, Technology & Work.

[CR22] Flemisch F, Kelsch J, Löper C, Schieben A, Schindler J, Waard D, Flemisch F, Lorenz B, Oberheid H, Brookhuis K (2008). Automation spectrum, inner/outer compatibility and other potentially useful human factors concepts for assistance and automation. Human Factors for Assistance and Automation.

[CR23] Goodall NJ (2016). Away from trolley problems and toward risk management. Applied Artificial Intelligence.

[CR24] Gürses, S. (2020). How Human-Machine Interaction keeps pace with automated vehicles: a systematic review. MSc thesis. Delft University of Technology https://repository.tudelft.nl/islandora/object/uuid%3A7ddf2758-577c-4e0f-98c6-54a20e45996d. Accessed 18 Dec 2020.

[CR25] Hancock PA (2020). Months of monotony – moments of mayhem: Planning for the human role in a transitioning world of work. Theoretical Issues in Ergonomics Science.

[CR27] Heikoop, D. D., Rodríguez Sayrol, A. & Hagenzieker, M. P. (2020a). Big Five Inventory-based participant selection calculation method. International Conference on Traffic and Transport Psychology (ICTTP), Goteborg, Sweden. (extended to 2022).

[CR26] Heikoop, D. D., Srinivasan Ravi Kumar, G. K., van Binsbergen, A. J., & Hagenzieker, M. P. (2020b). Personality and trust in automated cars: A correlation study. International Conference on Traffic and Transport Psychology (ICTTP), Goteborg, Sweden. (extended to 2022).

[CR238] Heikoop, D. D., Calvert, S. C., Mecacci, G., & Hagenzieker, M. P. (2020c). A practitioner’s view of driver training for automated driving from driving examiners: A focus group discussion. In 2020 Forum on Integrated and Sustainable Transportation Systems (FISTS) (pp. 14–19). IEEE.

[CR29] Heikoop DD, Hagenzieker MP, Mecacci G, Calvert SC, Santoni de Sio F, van Arem B (2019). Human behaviour with automated driving systems: A quantitative framework for meaningful human control. Theoretical Issues in Ergonomics Science.

[CR30] Hevelke A, Nida-Rümelin J (2014). Responsibility for crashes of autonomous vehicles: An ethical analysis. Science and Engineering Ethics.

[CR31] Himmelreich J (2019). Ethics of technology needs more political philosophy. Communications of the ACM.

[CR32] Van den Hoven, J. (2007), ICT and Value Sensitive Design, In: The Information Society: Innovation, Legitimacy, Ethics and Democracy In Honor of Professor Jacques Berleur S.j., edited by Philippe Goujon, Sylvian Lavelle, Penny Duquenoy, Kai Kimppa, and Véronique Laurent, 67–72. IFIP International Federation for Information Processing 233. Springer US.

[CR33] Human Right Watch. (2015). Mind the Gap: The Lack of Accountability for Killer Robots.

[CR34] Ibañez-Guzman J, Laugier C, Yoder JD, Thrun S (2012). Autonomous driving: Context and state-of-the-art. Handbook of Intelligent Vehicles.

[CR35] JafariNaimi N (2018). Our bodies in the trolley’s path, or why self-driving cars must *not* be programmed to kill. Science, Technology, & Human Values.

[CR36] Körber M, Cingel A, Zimmermann M, Bengler K (2015). Vigilance decrement and passive fatigue caused by monotony in automated driving. Procedia Manufacturing.

[CR37] Kyriakidis M, de Winter JCF, Stanton N, Bellet T, van Arem B, Brookhuis K, Martens MH, Bengler K, Andersson J, Merat N, Reed N, Flament M, Hagenzieker M, Happee R (2019). A human factors perspective on automated driving. Theoretical Issues in Ergonomics Science.

[CR38] Lin, P. (2015). Why Ethics Matters for Autonomous Cars, In: Autonomes Fahren, edited by Markus Maurer, J. Christian Gerdes, Barbara Lenz, and Hermann Winner, 69–85.

[CR39] Liu H-Y (2017). Irresponsibilities, inequalities and injustice for autonomous vehicles. Ethics and Information Technology.

[CR40] Mackworth NH (1948). The breakdown of vigilance during prolonged visual search. Quarterly Journal of Experimental Psychology.

[CR218] Matthias, A. (2004). The responsibility gap: Ascribing responsibility for the actions of learning automata. *Ethics and Information Technology*, *6(*3), 175–183. 10.1007/s10676-004-3422-1

[CR41] Manser, M. P., Noble, A. M., Machiani, S. G., Shortz, A., Klauer, S. G., Higgins, L., & Ahmadi, A. (2019). Driver training research and guidelines for automated vehicle technology, Technical Report No. 01–004. Department of Transport, TX. doi:10.13140/RG.2.2.31237.50401

[CR42] Marfoglia, T. (2020). The influence of take-over requests on driver workload: The role of personality - A driving simulation self-experiment. MSc thesis. Delft University of Technology, Delft, The Netherlands. https://repository.tudelft.nl/islandora/object/uuid%3Ad4a53002-3f50-4e4f-af84-62424d8ff15c. Accessed 18 Dec 2020.

[CR43] McKnight, A James, & Bert B Adams. (1970). Driver Education Task Analysis. Volume II: Task Analysis Methods. Final Report.

[CR44] Mecacci G, Santoni de Sio F (2020). Meaningful human control as reason-responsiveness: The case of dual-mode vehicles. Ethics and Information Technology.

[CR45] Merriman SE, Plant KL, Revell KMA, Stanton NA (2021). Challenges for automated vehicle driver training: A thematic analysis from manual and automated driving. Transportation Research Part F: Traffic Psychology and Behaviour.

[CR236] Michon, J. A. (1985). A critical view of driver behavior models: what do we know, what should we do? In Human behavior and traffic safety (pp. 485-524). Springer, Boston, MA.

[CR46] Milakis D, van Arem B, van Wee B (2017). Policy and society related implications of automated driving: A review of literature and directions for future research. Journal of Intelligent Transportation Systems.

[CR223] Mittelstadt, B. D., Allo, P., Taddeo, M., Wachter, S., & Floridi, L. (2016). The ethics of algorithms: Mapping the debate. *Big Data & Society*, *3*(2), 1–21. 10.1177/2053951716679679

[CR47] Mladenovic MN, McPherson T (2016). Engineering social justice into traffic control for self-driving vehicles?. Science and Engineering Ethics.

[CR48] Nihlén Fahlquist J (2009). Saving lives in road traffic—ethical aspects. Zeitschrift Fur Gesundheitswissenschaften.

[CR49] Norman WT (1964). Toward an adequate taxonomy of personality attributes: Replicated factor structure in peer nomination personality ratings. Journal of Abnormal and Social Psychology.

[CR214] Noto La Diega, G. (2018). Against the dehumanisation of decision-making. Algorithmic decisions at the crossroads of intellectual property, data protection, and freedom of information. *Journal of Intellectual Property, Information Technology and Electronic Commerce Law*. 10.31228/osf.io/s2jnk

[CR50] Nyholm N (2018). The ethics of crashes with self-driving cars: A roadmap. Philosophy Compass.

[CR222] Pagallo, U. (2013). The Laws of Robots: Crimes, Contracts, and Torts. Springer.

[CR51] Parasuraman R, Sheridan TB, Wickens CD (2000). A model for types and levels of human interaction with automation. IEEE Transactions on Systems, Man, and Cybernetics-Part a: Systems and Humans.

[CR52] Rasmussen J (1983). Skills, rules, and knowledge; signals, signs, and symbols, and other distinctions in human performance models. IEEE Transactions on Systems, Man, and Cybernetics.

[CR53] Russell, S. (2019). Human Compatible: Artificial Intelligence and the Problem of Control. Viking. US. ISBN 978-0-525-55861-3

[CR54] SAE (2018). Taxonomy and definitions for terms related to on-road motor vehicle automated driving systems.

[CR55] Sanchez, F., Blanco, R. & Luis Diez, J. (2016). "Better together: cooperative technologies will be vital to the development of highly autonomous vehicles operating in complex urban environments." Vision Zero International.

[CR56] Santoni de Sio F, Mecacci G (2021). Four responsibility gaps with artificial intelligence: Why they matter and how to address them. Philosophy Technology.

[CR57] Santoni de Sio F, Van den Hoven J (2018). Meaningful human control over autonomous systems: a philosophical account. Front. Robot. AI.

[CR225] Santoro, M., Marino, D., & Tamburrini, G. (2008). Learning robots interacting with humans: from epistemic risk to responsibility. *AI & SOCIETY*, *22*(3), 301–314. 10.1007/s00146-007-0155-9

[CR58] Shladover SE (2018). Connected and automated vehicle systems: Introduction and overview. Journal of Intelligent Transportation Systems.

[CR216] Simpson, T. W., & Müller, V. C. (2016). Just War and Robots’ Killings. *The Philosophical Quarterly*, *66*(263), 302–322. 10.1093/pq/pqv075

[CR220] Sparrow, R. (2007). Killer Robots. *Journal of Applied Philosophy*, *24*(1), 62–77. 10.1111/j.1468-5930.2007.00346.x

[CR210] Stilgoe, J. (2017). Machine learning, social learning and the governance of self-driving cars. *Social Studies of Science*. 10.1177/0306312717741687 10.1177/0306312717741687 29160165

[CR211] Stilgoe, J., Owen, R., & Macnaghten, P. (2013). Developing a framework for responsible innovation. Research Policy, *42*(9), 1568–1580. 10.1016/j.respol.2013.05.008

[CR59] Struik A (2021). Meaningful Human Control over Automated Driving Systems: Driver intentions and ADS behaviour.

[CR217] Tigard, D. W. (2020). There Is No Techno-Responsibility Gap. *Philosophy & Technology* 2020 34:3, *34*(3), 589–607. 10.1007/S13347-020-00414-7

[CR60] Theologus, George C. & Fleishman E. A. (1971). "Development of a taxonomy of human performance: Validation study of ability scales for classifying human tasks." In.: American institutes for research Pittsburgh PA.

[CR229] Vellinga, N. E. (2019). Automated driving and its challenges to international traffic law: which way to go? *Law, Innovation and Technology**11*(2), 257–278. 10.1080/17579961.2019.1665798

[CR215] Wachter, S., Mittelstadt, B., & Floridi, L. (2017). Why a Right to Explanation of Automated Decision-Making Does Not Exist in the General Data Protection Regulation. *International Data Privacy Law*, *7*(2), 76–99. 10.1093/idpl/ipx005

[CR61] Waymo (2020), Waymo Safety Report September 2020, https://waymo.com/safety/ (accessed 6^th^ Jan 2021).

[CR62] Wiener EL (1985). Beyond the sterile cockpit. Human Factors.

[CR63] Young MS, Stanton NA, Harris D (2007). Driving automation: Learning from aviation about design philosophies. International Journal of Vehicle Design.

[CR64] Zahabi M, Razak AMA, Mehta RK, Manser M (2021). Effect of advanced driver-assistance system trainings on driver workload, knowledge, and trust. Transportation Research Part F: Traffic Psychology and Behaviour.

